# A second chance for first impressions: evidence for altered impression updating in borderline personality disorder

**DOI:** 10.1186/s40479-024-00259-y

**Published:** 2024-07-18

**Authors:** Kevin Konegen, Georg Halbeisen, Georgios Paslakis

**Affiliations:** University Clinic for Psychosomatic Medicine and Psychotherapy, Medical Faculty, Campus East-Westphalia, Ruhr-University Bochum, Virchowstr. 65, 32312 Luebbecke, Germany

**Keywords:** Borderline personality disorder, Interpersonal problems, Social cognition, Belief updating, Renewal, Impression formation, Attitudes, Psychotherapy

## Abstract

**Background:**

Individuals with borderline personality disorder (BPD) frequently alter between idealizing and devaluing other persons, which has been linked to an increased tendency to update self-relevant beliefs and impressions. We hypothesized that increased impression updating could stem from reduced attitude contextualization, i.e., a process in which impression-disconfirming information is linked to contextual cues.

**Methods:**

Individuals diagnosed with BPD and controls (recruited online, with unknown diagnostic status) completed an impression formation paradigm. They first learned about the positive or negative behaviors of others in one Context A (e.g., Person 1 is helpful), followed by learning about behaviors of the opposite valence in a second Context B (Person 1 is rude). We also manipulated between participants whether the observed behaviors were directed toward the study participants (self-relevant) or, more generally, at other people (other-relevant). The contexts were marked by differently-colored backgrounds (e.g., yellow vs. blue), to avoid influences of prior knowledge or experiences. After exposure to information in both contexts, participants rated their impressions of the persons in Context A, Context B, and, crucially, a previously unknown Context C (white background). We examined whether the initial or an updated impression (re-)emerged in Context C.

**Results:**

Initial impressions remained stable and dominated the ratings of controls across contexts A, B, and C for both self-relevant and other-relevant behaviors, consistent with contextualizing impression-disconfirming information. As expected, however, individuals with BPD only showed updated impression ratings in Context C for self-relevant behaviors, consistent with the assumed reduced tendency to contextualize impression-disconfirming self-relevant information. Further exploratory analyses suggest that more severe BPD symptoms predicted more pronounced impression updating in the self-relevant condition.

**Conclusions:**

The findings help to illuminate the mechanisms underlying interpersonal problems in individuals with BPD. People with BPD are not just more inclined to discard positive first impressions but to re-evaluate disliked others when they behave positively, contributing to the volatility of interactions with others. Contextualization has known and modifiable antecedents, and the study may thus provide potential targets for therapeutic intervention. Future studies will need to replicate the findings with specified controls.

**Supplementary Information:**

The online version contains supplementary material available at 10.1186/s40479-024-00259-y.

## Background

Borderline personality disorder (BPD) is a severe mental disorder that affects about 0.7–2.7% of the general population [1, 2]. BPD is associated with high rates of comorbid mood and anxiety disorders, substance abuse, and disordered eating [3, 4] and poses an increased risk for self-injury and suicidal behaviors [5]. A core and diagnostic feature of BPD are interpersonal problems, which cause a significant amount of burden [6] and contribute to dysfunctional social behaviors and suicide risk [7, 8]. Interpersonal problems in BPD include a pattern of unstable and intense relationships that are marked by alternating between extreme idealization and devaluation of the other person [9]. Given the associated burden and central role of social functioning, it is an important research goal to illuminate which processes contribute to interpersonal problems in individuals with BPD [10].

Recent studies suggest that alterations in impression formation, i.e., the processes involved in perceiving and making sense of others, contribute to interpersonal problems in individuals with BPD—among other factors such as pronounced emotional dysregulation (i.e., the inability to respond to and manage emotions flexibly), impulsivity, and hyper-mentalization (i.e., the over-attribution of intentions and emotions to oneself and others) [11]. For example, individuals with BPD tend to form negative impressions when first encountering others [12, 13] and show increased memory for negative person-related attributes [14]. A propensity for negative impressions likely impairs relationship building, as impressions, often formed unintentionally and from limited information [15, 16], may negatively bias how one processes and interprets a person’s attributes or ambiguous behaviors [17–19].

Besides negativity biases in impression formation, individuals with BPD may also show alterations in updating impressions when faced with novel, “disconfirming” information. People often behave inconsistently, and initial positive impressions could thus be disconfirmed by people acting, e.g., rude or hostile on a second occasion; vice versa, initial negative impressions could be disconfirmed by people later acting friendly or helpful. In individuals without BPD, initial positive and negative impressions often remain remarkably stable over time [20, 21] and even when faced with disconfirming information [22], suggesting slow rates of updating impressions once created. This inertia is mainly functional, as initial impressions are demonstrably accurate [23] and help to guide approach and avoidance behavior in complex interactions [24].

Individuals with BPD, however, are known to swiftly and frequently alter between, e.g., first idealizing and then devaluing another person, which implies their initial impressions are highly malleable. Kube and Rozenkrantz [25] attributed this pattern to a more pronounced and general tendency in individuals with BPD to update self-relevant beliefs when faced with disconfirming information. A study on social feedback processing found that individuals with BPD integrated undesirable social feedback into their self-impressions to a greater extent than individuals without BPD, but there were no differences in impression updating for feedback directed at (self-irrelevant) others [26]. This swift integration of new information into self-relevant beliefs could, therefore, aide in explaining behavioral inconsistencies in social interactions and interpersonal problems in individuals with BPD [25].

Thus far, there are only a few explanations why individuals with BPD would show increased impression updating. For example, increased updating could result from an overall low confidence in one’s beliefs or an overreliance on external feedback [27]. Here, we propose and investigate that increased impression updating could stem from aberrant *attitude contextualization*. According to the Representational Account of attitude change [28], initial impressions often remain stable because people contextualize “counter-attitudinal”, i.e., disconfirming information. An initial positive or negative encounter is assumed to create a memory trace that directly links the encountered person to a positive or negative impression in a context-free, generalized representation. This impression will be activated upon re-encountering the person (“Ah, here comes Dave, what a nice person!”). Should the person then behave counter-attitudinally and violate behavioral expectations, attention will shift toward contextual cues in search of an explanation (“Why is Dave suddenly unpleasant?”). As a result, the person, the cue, and the (discrepant) impression will be linked in a novel, now contextualized representation instead of replacing or amending the initial impression (“Dave is unpleasant when it’s raining”). Thus, the novel impression (“unpleasant”) will be activated when re-encountering the person in that context (“raining”), but the initial impression (“nice”) will prevail in the original or any other context [29]. Note that in laboratory studies, contextualization patterns in impression formation are usually investigated with artificial, “evaluatively neutral” instead of more ecologically valid contexts (e.g., colored backgrounds instead of specific situations) to avoid influences of prior knowledge or experiences on attitude contextualization [29, 30]. This line of research, inspired by human fear and extinction learning [31], has proven extremely valuable for explaining heterogeneity in the stability and malleability of impressions by considering the context in which novel information is encountered [32].

Individuals with BPD often fear being rejected [33], being accepted [34, 35], they mistrust others [36], and expect higher behavioral volatility [37]. Because contextualization requires an expectation of behavioral consistency, we therefore assumed that individuals with BPD show decreased contextualization tendencies, especially for self-relevant impressions. Being appreciated and then turned away (or vice versa) might not trigger an expectancy violation in individuals with BPD that draws attention to context, thus preventing the formation of a contextualized impression. In addition, individuals with BPD show impairments in attentional control [38, 39], which could further affect their ability to attend to contextual cues selectively. We would, therefore, expect individuals with BPD to update their initial impressions when faced with disconfirming information instead of forming contextualized impressions, that is, to alter the valence of the interpersonal impressions.

### The present research

The present study aimed to test whether individuals with BPD, compared to controls, show decreased tendencies to contextualize impressions. For this purpose, we adapted a previously used impression formation paradigm which included a learning task, an evaluation task, and different artificial contextual cues [28, 30, 40]. Participants would first learn about the positive or negative behaviors of others in one context (Context A, e.g., *Dave is helpful*), followed by learning about their behaviors of the opposite valence in a second context (Context B, e.g., *Dave is rude*). Afterwards, participants would rate their impressions of the persons in Context A, Context B, and, crucially, in a previously unknown Context C.

Based on the Representational Account [28], we expected controls to form a generalized impression initially, with exposure to disconfirming information triggering contextualization. Thus, the impressions assessed in Contexts A and B should be sensitive to the valence of information encountered in these contexts, and, critically, the impression in the novel Context C should be consistent with the valence of the *initially* presented information. In other words, contextualization in controls would predict that the initial impression remains stable and re-emerges in Context C (i.e., liking Dave).

For individuals with BPD, however, we would expect decreased impression contextualization, and that the initial impression formed in Context A is updated in Context B. Thus, we expected the impression in the novel Context C to be consistent with the valence of the *most recently* presented information. In other words, the updated rather than the initial impression should manifest in Context C (i.e., disliking Dave; a similar effect could be predicted for Context B, but see Discussion). Because previous research suggests that altered impression updating could specifically concern self-relevant cognitions [25], we additionally manipulated between participants whether the observed behaviors were directed toward the study participants (the “self”) or, more generally, at other people (the “other”). We expected more pronounced differences in impression contextualization between individuals with BPD and controls for self-relevant compared to other-relevant behaviors.

## Methods

### Participants, design, and setting

We recruited adults diagnosed with BPD and controls for an experiment with a 2 (group: BPD vs. controls) × 2 (relevance: self vs. other) × 2 (initial valence: positive vs. negative) mixed-measures design, with relevance manipulated between and valence manipulated within participants. Individuals for the BPD group were recruited locally among inpatients who received Dialectical Behavior Therapy at the University Clinic for Psychosomatic Medicine and Psychotherapy, Luebbecke, Germany. The manualized therapy is typically completed within 6 to 8 weeks. We only included adult inpatients (18 years or older) with a validated BPD diagnosis based on the results of a structured clinical interview according to DSM-IV criteria [41], which was conducted by the clinic’s trained physicians and clinical psychologists after admission. Controls were recruited online from university and social network forums and among colleagues, friends, and acquaintances. We only included adults and excluded those as controls who indicated a history of BPD or currently receiving (any) psychotherapeutic treatment.

We targeted a sample of *N* = 100 (50 patients, 50 controls). Our initial power calculation with G*Power [42] suggested *n* = 50 per group would achieve a power of 1-*β* = 0.80 to detect a medium-sized difference (d = 0.50) between BPD vs. control participants at α = 0.05 (one-tailed), for example, in a between groups *t*-test of impression scores within a specific context. However, we calculated a posteriori that the sample also had similar power to detect a within-between interaction of f = 0.25 in the reported MANOVA with 4 groups and 6 measurements. The study was reviewed and approved by the Ethics Committee of the Ruhr-University Bochum’s Medical Faculty at Campus East-Westphalia (AZ 2021 − 790_3, February 10th, 2022), prospectively registered at https://aspredicted.org/X1L_XDY, and conducted in accordance with the Declaration of Helsinki. All participants gave informed consent. Data collection lasted from February 2022 to July 2023. We did not conduct any interim analysis and report all measures, manipulations, and exclusions. Data and materials can be obtained from the corresponding author upon request.

### Measures and procedure

We implemented the study online in jsPsych [43]. Study participation required a physical keyboard. Inpatients participated during their stay using a laptop provided locally by the experimenter; controls participated remotely using a keyboard-based device at their disposal. Upon starting the study, participants were randomly assigned to self-relevant or other-relevant conditions and read the study information and consent forms. After providing informed consent, we assessed participants’ age, gender, German language fluency, years of education, history of BPD, current psychotherapeutic treatment, and previous study participation. The latter question (together with a self-generated identifier) served to exclude datasets from repeated participations of controls participating remotely.

We then assessed, in randomized order, BPD symptoms using the short version of the Borderline Symptom List (BSL-23) [44], and rejection sensitivity using the short version of the Rejection Sensitivity Questionnaire (RSQ-9) [45, 46]. The BSL-23 assessed experiences typically reported by patients with BPD within the prior week on a 5-point scale (from 0, not at all, to 4, very strong), with the mean score across items serving as an index of BPD-specific symptom severity [47]. The RSQ-9 asked participants, across nine hypothetical rejection scenarios, how concerned they would be if rejected (from 1, not at all, to 6, very concerned), and if they expect being accepted (i.e., not rejected; from 1, very unlikely, to 6, very likely). For each item, concern is multiplied by the expectation of rejection (i.e., the reversed acceptance rating), with the sum across items serving as an index of how negatively one reacts to subtle cues of rejection. We included these questionnaires to explore whether impression updating tendencies were associated with BPD symptoms or enhanced rejection sensitivity.

#### Impression learning task

**Fig. 1:**
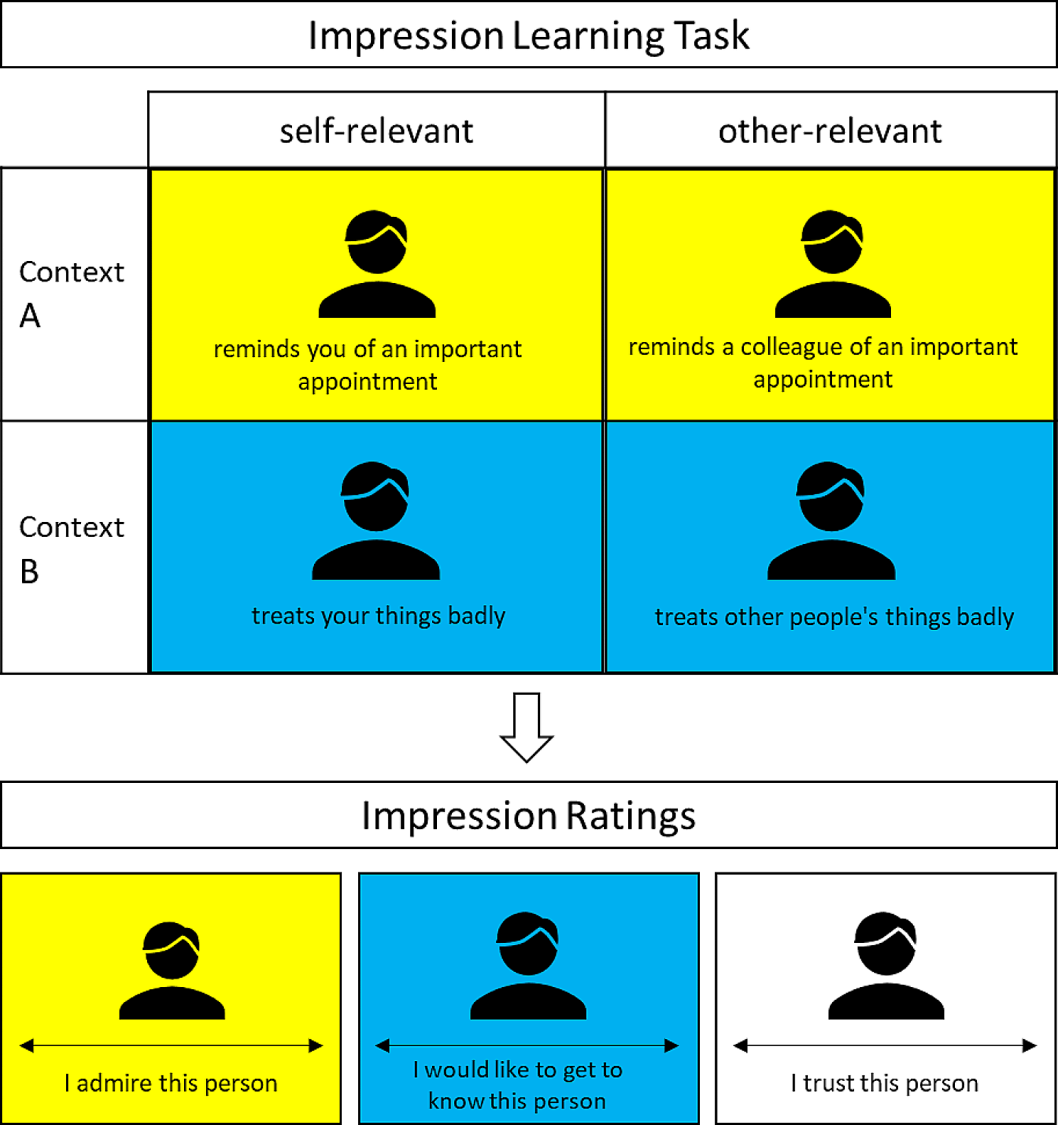
Illustration of the study procedure. Participants were asked to imagine meeting new people for the first time, and to observe how these individuals behaved (top panel). To examine impression contextualization or updating after exposure to disconfirming information, participants next rated their impressions of the four people in different contexts in randomized order (lower panel)

After completing the initial assessments described above, participants commenced the evaluative learning task with evaluatively neutral contexts as used in previous research [28, 30, 40]. Participants were asked to imagine meeting new people for the first time (e.g., at work or privately), and to observe how these individuals behaved (see top panel Fig. 1). Specifically, we asked: “We would like to ask you to read various descriptions of people’s behaviors. Please imagine that you are meeting these people for the first time (e.g., at work or in your private environment) and are now gradually observing how they behave. The descriptions of the behaviors are shown to you one after the other and are always presented for a few seconds. Please read each description carefully.” Each trial showed a previously used, black-and-white photograph of one of four men’s faces with neutral expression [40, 48]. We chose men’s faces only to avoid increasing the complexity of the design, and because feminine facial features have been shown to elicit liking and thus to be less neutral [49].

Each face was shown with four statements describing a positive and four statements describing a negative behavior directed at the participant (e.g., “reminds you of an important appointment”) or another person (“reminds a colleague of an important appointment”), depending on the assigned between-participants condition (we manipulated relevance between participants to avoid cross-contamination between the conditions, e.g., participants taking all behaviors personal). The 32 statements were shown only once, were adapted from Rydell and Gawronski [30], and selected based on a pilot test, such that valence ratings of positive and negative statements were comparable between self- and other-relevant versions (see Additional file 1). We asked participants to read each statement carefully. Face-statement pairs were presented for 6000 ms with an inter-trial interval of 1000 ms.

Critically, the learning task was divided into two blocks that were marked by differently colored backgrounds. These colors, devoid of a specific meaning or learned association with other stimulus materials, have been previously used to critically test the idea that exposure to disconfirming information—and not to a specific context—triggers contextualization processes [32]. Thus, the first block of 16 trials was marked randomly by either a blue or yellow background (Context A), and paired each of two faces with four positive statements, and each of the other two faces with four negative statements. The second block of 16 trials, marked by the previously unchosen yellow or blue background (Context B), reversed the pairing such that the faces initially associated with positive statements were each shown with four negative statements, and the faces initially associated with negative statements were each shown with four positive statements. Thus, after forming an impression in Context A, participants were always exposed to disconfirming information in Context B (both positive to negative and negative to positive). The assignment of faces to statements and contexts, and the order within contexts, were fully randomized for each participant.

#### Impression ratings

To examine impression contextualization or updating after exposure to disconfirming information, participants next rated their impressions of the four people in different contexts (see lower panel Fig. 1). The ratings were always given only after exposure to information in both contexts A and B, which is the established procedure [29], as interim evaluations by themselves might potentially demarcate a context change that could affect subsequent evaluations or influence evaluative strategies that could conceal group differences [50, 51] (but please see Discussion). Each photo was rated once in front of the initial Context A (e.g., blue background), the disconfirming Context B (e.g., yellow background), and a novel Context C (i.e., white background), such that there were two ratings per context and initial valence condition (overall 12 ratings). The presentation order of photos and contexts was fully randomized for each participant. Ratings were provided on a visual analogue scale (VAS) with an internal range from 0 (not at all) to 200 (completely), indicating how much participants agreed with a favorable person description (e.g., I admire this person; I would like to get to know this person). We used 12 different descriptions, adapted from the Interpersonal Liking scale [52] and Rubin’s liking scale [53], which were randomly assigned toward the persons and conditions per each participant. After completing all ratings, participants were thanked and dismissed.

### Data aggregation and analysis

We extracted and averaged impression ratings per context and initial valence condition for each participant and submitted them to a 2 (group: BPD vs. controls) × 2 (relevance: self vs. other) × 2 (initial valence: positive vs. negative) multivariate analysis of variance (MANOVA)^1^. The averaged impression ratings per valence condition obtained in the three different contexts A, B, and C served as the MANOVA’s dependent variables. We followed-up on significant interactions with repeated-measures ANOVAs for each context.

The questionnaires showed high levels of internal consistency (Cronbach’s α = 0.97, 0.91, for the BSL and RSQ-9, respectively) and were thus aggregated according to their convention. We compared these scores between groups with independent samples *t*-tests; *t*-tests and χ² frequency tests were used to compare participant characteristics. We further used the questionnaire scores to explore whether impression contextualization vs. updating tendencies were associated with BPD symptoms or rejection sensitivity in moderation and mediation models.

The significance level for all analyses was set at *p* ≤ .05. Post hoc pairwise comparisons report Bonferroni-adjusted *p*-values for multiple comparisons. Effect sizes are reported as *η*_*p*_*²*. Variable values are reported as means and standard deviations (SDs). The data were aggregated and analyzed with IBM SPSS 28 [54]. We used PROCESS v4.2 [55] with boot-strapped (5,000 samples) bias-corrected 95% confidence interval (CI) to explore moderation and mediation patterns.

## Results

### Sample characteristics

We collected *N* = 104 datasets. We excluded two inpatients who did not fulfill the diagnostic criteria, five controls who indicated they had a BPD diagnosis or currently received psychotherapeutic treatment, and five datasets from repeated participations, leaving *N* = 92, with *n* = 46 patients with BPD (35 women, 7 men, 4 other; *M*_*age*_ = 26.4, age range: 18–52 years), and *n* = 46 controls (37 women, 7 men, 2 other; *M*_*age*_ = 29.4, age range: 19–61 years). The groups did not differ in terms of age, gender composition, or German language fluency, but patients with BPD, similar to other studies [56], reported fewer years of school attendance. Consistent with their diagnosis, patients with BPD had higher BSL and RSQ scores than controls (see Table 1).

**Table 1:** Participant sociodemographic descriptive statistics (mean with SD in parenthesis,* or n)*

Parameter	Total	BPD	Control
n	92	46	46
age	27.9 (10.2)	26.4 (8.5)	29.4 (11.7)
gender			
women	72	35	37
men	14	7	7
other	6	4	2
German language fluency			
first language	83	42	41
fluent	9	4	5
education			
12 years or more	69	30	39
less than 12 years	23	16	7
BSL-23	1.4 (1.1)	2.3 (0.7)	0.4 (0.4)
RSQ	123.3 (63.4)	168.2 (53.1)	78.5 (34.6)

Note. BPD = Borderline Personality Disorder; BSL-23 = Borderline Symptom List mean score; RSQ = Rejection Sensitivity Questionnaire sum score

### Context effects on impression ratings

We hypothesized that patients with BPD, compared to controls, show decreased contextualization of impressions that are inconsistent with previous experiences. The overall 2 (group) × 2 (relevance) × 2 (initial valence) MANOVA with impression ratings across the three contexts as dependent variables found a significant main effect of valence, *F*(3, 86) = 4.68, *p* = .004, Wilk’s Λ = 0.86, *η²* = 0.14, and group, *F*(3, 86) = 4.99, *p* = .003, Wilk’s Λ = 0.85, *η²* = 0.15, qualified by a valence × group interaction, *F*(3, 86) = 6.68, *p* < .001, Wilk’s Λ = 0.81, *η²* = 0.19, which was further qualified by a valence × group × relevance interaction, *F*(3, 86) = 3.41, *p* = .02, Wilk’s Λ = 0.89, *η²* = 0.11. The relevance main effect and valence × relevance interaction were not significant, *F*(3, 86) = 0.6, *p* = .61 and *F*(3, 86) = 0.1, *p* = .95, respectively. To specify the three-way interaction across contexts in terms of our hypothesis, we conducted three separate 2 (group) × 2 (relevance) × 2 (valence) follow-up ANOVAs for the ratings obtained in contexts A, B, and C, respectively.

#### Initial context A

**Fig. 2:**
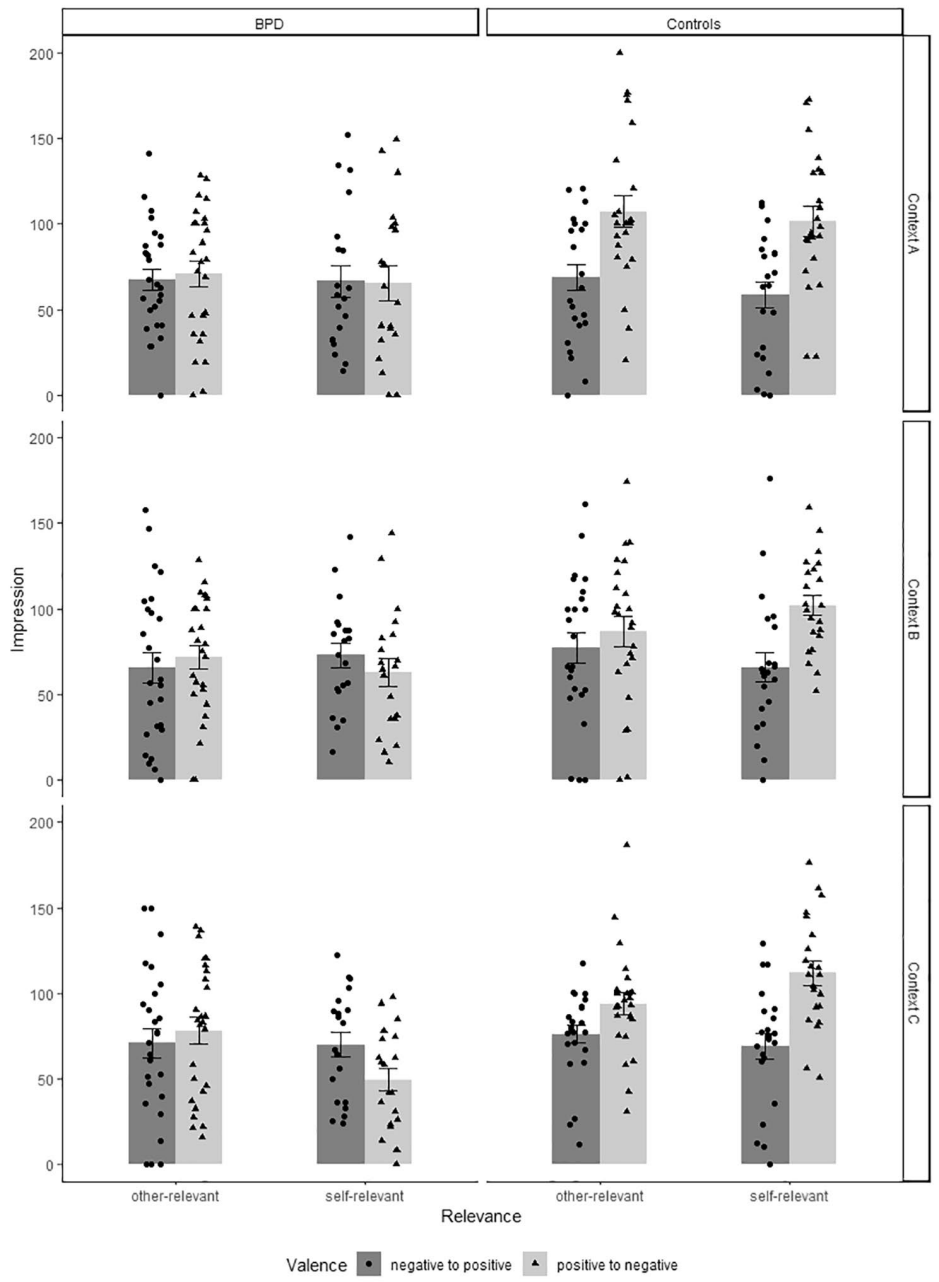
Impression rating means in the first (**A**), second (**B**), and novel (**C**) contexts as a function of group (BPD vs. controls), relevance (self vs. other), and valence change (positive to negative vs. negative to positive). Error bars show standard error of the mean

Impression ratings in the first learning context (Fig. 2a) varied by valence, *F*(1, 88) = 11.28, *p* < .001, *η²* = 0.11, group, *F*(1, 88) = 9.39, *p* = .003, *η²* = 0.10, and a group × valence interaction, *F*(1, 88) = 9.92, *p* = .002, *η²* = 0.10; all other *F*s < 1.1, *p*s > .31. Controls evaluated faces in Context A consistent with the valence of initial statements, with faces initially paired with positive statements rated more likeable than faces initially paired with negative statements, *p* < .001. Ratings by patients with BPD did not differ based on initial valence, *p* = .88.

#### Disconfirming context B

Impression ratings in the second learning context (Fig. 2b) varied by group, *F*(1, 88) = 5.76, *p* = .02, *η²* = 0.06, and a group × valence interaction, *F*(1, 88) = 5.28, *p* = .02, *η²* = 0.06; all other *F*s < 3.8, *p*s > .05. Controls still preferred the faces initially paired with positive statements over initially negatively paired faces, *p* = .003, but at only half the effect size compared to Context A (effect sizes from post hoc comparisons: *η²* = 0.09 vs. 0.20, respectively), consistent with the assumed contextualization of disconfirming information. Patients with BPD showed no preference, *p* = .80.

#### Novel context C: stability vs. updating

Evaluations in the third novel Context C (Fig. 2c) varied by valence, *F*(1, 88) = 6.84, *p* = .01, *η²* = 0.07, group, *F*(1, 88) = 13.64, *p* < .001, *η²* = 0.13, a group × valence interaction, *F*(1, 88) = 16.57, *p* < .001, *η²* = 0.16, and the expected group × valence × relevance interaction, *F*(1, 88) = 8.44, *p* = .005, *η²* = 0.09; all other *F*s < 3.4, *p*s > .07. Post hoc comparisons within the three-way interaction showed that controls evaluated faces that were initially paired with positive statements more positively than faces initially paired with negative statements, for both self-relevant and other-relevant conditions, *p*s < .001 and .05. In other words, the initial impression re-emerged in Context C, regardless of relevance. However, patients with BPD showed a reversed pattern, preferring the faces initially paired with negative statements over initially positively paired faces in the self-relevant condition, *p* = .04. In other words, the impression ratings reflected the information given in the second Context B rather than A, consistent with the assumed updating of the initial impressions in patients with BPD. Ratings in the other-relevant condition were descriptively congruent with the information provided in the initial Context A, but the difference was not statistically significant, *p* = .39.

### Exploratory analysis

**Fig. 3:**
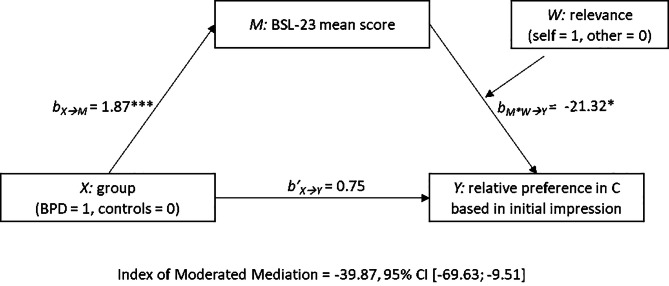
A moderated mediation model with group as predictor *X* (coded 1 0 for BPD, controls), BSL-23 symptom scores as mediator *M*, relative preferences in Context C as criterion *Y*, and relevance as moderator *W* (coded 1 0 for self, other). The boot-strapped model confirmed the effect via BPD symptom severity on impression updating in Context C, but only in the self-relevant condition

Since the results largely supported the hypothesized increased impression updating tendencies in individuals with BPD for self-relevant information, we further explored associations between impressions in Context C and BPD symptoms. We computed differences scores between ratings for initially positively and initially negatively paired faces within Context C, such that positive vs. negative values reflected the relative stability vs. updating of initial impressions, respectively. We reasoned that the observed interaction between group and relevance on relative preferences in Context C should be driven by the severity of BPD symptoms, and thus explored a moderated-mediation pattern with group as predictor *X* (coded 1 0 for BPD, controls), BSL-23 symptom scores as mediator *M*, relative preferences in Context C as criterion *Y*, and relevance as moderator *W* (coded 1 0 for self, other; see Fig. 3). This analysis showed that group predicted symptoms scores, *b*_*X◊M*_ = 1.87, *p* < .001, which, in turn, interacted with relevance to predict relative preferences in Context C, *b*_M*W◊Y_ = -21.31, *p* = .01, *R²*_*Change*_ = 0.06. Simple slopes showed that, in the other-relevant condition, symptom scores exerted no effect, *b*_*M◊Y*_ = 0.75, *p* = .93. However, in the self-relevant condition, more severe BPD symptoms predicted more pronounced impression updating, *b*_*M◊Y*_ = -20.57, *p* = .01, as indicated by the negative sign of the regression weight. The boot-strapped model confirmed the pattern, as the index of moderated mediation (IMM) was significant, *IMM* = -39.86, 95% CI [-69.63; -9.51]. We could not identify a similar pattern for the rejection sensitivity scores, or when using the relative preferences within Context A or within B as criteria.

## Discussion

This study investigated the stability vs. updating of impressions in individuals with BPD who were faced with impression-disconfirming information. Participants with BPD and controls completed an adapted impression formation task in which they would first learn about the positive or negative behaviors of others in one context (Context A), followed by learning about the others’ behaviors of the opposite valence in a second context (Context B). Afterwards, participants rated their impressions of the displayed persons in Context A, Context B, and, crucially, in a previously unknown Context C. For controls, ratings remained in line with the valence of the initial information in the first Context A, to a lesser extent in the second Context B, and crucially, re-emerged in the new Context C, consistent with the idea that controls’ impressions remain stable due to a tendency to contextualize disconfirming information. For individuals with BPD, however, we only observed impressions in the novel Context C in line with the valence of the most recently presented, i.e., disconfirming information. Note that this effect is only attributable to the manipulated valence of the statements, as the identical persons were evaluated in all three contexts. This suggests that the disconfirming information updated the initial impressions rather than being contextualized. As in previous studies [25], impression updating only occurred for self-relevant information and was linked to the severity of BPD symptoms, suggesting a high specificity of the decreased contextualization tendencies (for further support, we report a re-analysis of the data with BPD symptoms as a continuous rather than categorical predictor, see Additional file 2).

Should we have expected an updated, context-free impression in individuals with BPD to also manifest in the evaluations in Context B? The evaluative patterns in contexts B and C were indeed descriptively similar, but a significant effect only emerged for evaluations in Context C. The Representational Account [28] assumes that contexts, when linked to impressions in a contextualized representation, merely constrain the activation of evaluative information about a target person without becoming meaningful themselves; thus, entirely ignoring contexts and updating the initial impression would predict similar evaluations in contexts B and C. However, although meta-analytic evidence confirms the constraining function of contexts, it also shows that contexts can become directly associated with the counter-attitudinal experience [29]. As a result, while the pattern in Context C likely reflects the unaltered effect of an updated impression, one could speculate that the muted pattern in Context B represents a joint influence of an updated impression and an acquired context meaning (e.g., the conflict could have rendered B aversive [57] or a signal of distrust [58]).

The overall findings may help to further illuminate the mechanisms underlying interpersonal problems in individuals with BPD. Kube and Rozenkrantz [25] emphasized that beliefs modulate how people perceive and respond to the world around them and that the tendency to rapidly update beliefs about others partly explains interpersonal problems in both healthy and clinical populations. Our findings suggest that, at least in individuals with BPD, increased updating could stem from failing to contextualize belief-disconfirming information, which is one mechanism that otherwise contributes to the stability of interpersonal impressions and safeguards against their rapid revisions [28]. Thus, people with BPD may not just be more inclined to perceive others negatively [12, 13] and discard positive first impressions; they appear to be equally prone to “embrace” previously hostile others when they behave positively, creating potential for future interpersonal conflicts. Indeed, although we could not identify other study data consistent with this observation, our clinical experiences corroborate that some people with BPD fear they are “gullible” or more easily influenced and exploited by others.

The process-based focus advocated by Kube and Rozenkrantz [25], and supported by our findings, is also compatible with the shift towards a dimensional conceptualization of personality disorders included in the newer versions of the Diagnostic and Statistical Manual of Mental Disorders (DSM-V; [9] and the International Statistical Classification of Diseases (ICD-11; [59]). In the DSM-V, diagnosing a borderline “type” personality disorder requires the presence of impairments in self- and interpersonal functioning combined with traits from domains such as negative affectivity, detachment, antagonism, disinhibition, and psychoticism [60]. Similarly, in the ICD-11, diagnosing a personality disorder entails a global evaluation of self- and interpersonal functioning, with a “borderline pattern specifier” defined by further similar trait features [61]. On a descriptive basis, we would expect the here observed effects to show close associations with the degree of interpersonal dysfunction (with corresponding ratings including items potentially linked to contextualization deficits such as “I often find it hard to tolerate it when others have a different opinion”; [62]). However, whether and how contextualization might be linked to more specific trait domains or might respond to or inform the development of trait-specific interventions [63], is highly speculative and requires additional investigation.

Still, since contextualization has known and modifiable antecedents [32], our study may suggest preliminary targets for therapeutic intervention, such as cognitive restructuring techniques to enhance contextualization skills and promote more adaptive social cognition in individuals with BPD. For example, we reasoned that individuals with BPD may simply not expect others to behave consistently [33–37], especially towards them, lowering the potential for expectancy violations which guide attention to context. However, expectancy violations are but one process relevant to drawing attention to context, and patients could also be taught to consider contextual explanations of the behavior of others or to direct their attention to situation cues and critically evaluate them whenever they perceive others negatively (i.e., without implying that all situations may explain negative behaviors by others). It is also possible that the observed group differences are linked to further cognitive processes besides expectations and attention control. For example, Herzog et al. [27] suggested linking updating processes to altered inhibitory control and reward processing. Further necessary investigations into the underlying processes of altered impression updating are thus likely to reveal more precise implications.

### Limitations

Of course, interpreting the study findings is subject to limitations. First, although our findings are consistent with explaining increased updating tendencies by lowered contextualization, we only inferred these processes from group-based comparisons and did not manipulate the implied processes directly. Thus, we are currently unaware whether individuals with BPD may fail to engage in contextualizing impressions or whether they may lack the capacity to contextualize in the first place. For example, it may be possible that individuals with BPD nevertheless contextualize inconsistent impressions in situations known to elicit different behaviors (e.g., when encountering a person under stress, or intoxicated), which would imply that contextualization is possible, but otherwise not initiated. A comparison of artificial and more ecologically valid contexts could thus be fruitful.

Second, and relatedly, we thus far only speculated why individuals with BPD fail to contextualize, citing missing expectancy violations and altered attentional control. We observed that a more general BPD severity score predicted the updating of impressions but not a measure of more specific expectations of social rejection. This could suggest that a mixture of processes accounts for the observed effects and that it is necessary to further specify the antecedent conditions of impression updating in future investigations. One could consider, for example, comparing contextualization processes between impression formation and completely non-social tasks (i.e., evaluative learning with objects instead of persons as targets) to estimate the contribution of alterations in social cognition compared to non-social processes.

Moreover, the generalizability of the findings may be limited by the characteristics of the sample. Our participants were primarily women and we recruited fewer participants than originally intended, and thus may have missed true but small effects, or overestimated the sizes of those effects detected. We did not assess co-morbidities in individuals with BPD (e.g., depression, or trauma-related disorders) or current medications (e.g., quetiapine or SSRIs), and our control group did not undergo a formal diagnostic assessment. We thus cannot exclude that the present findings, at least partly, were affected by further unknown health statuses of BPD or control participants. It is further important to note that controls participated online and remotely, potentially reducing data precision and leading to more selective samples. Data collection on online survey platforms is usually associated with a higher proportion of low-quality responses [64], especially for participants using study participation as a main source of income [65]. We neither incentivized study participation, nor collected data on survey platforms (or noticed implausible or outlying responses). Common and applicable issues of online data collection stem from non-probabilistic sampling (i.e., only individuals with internet access and within our extended social circles, with an interest and possible previous experience in study participation likely completed the study) and the lack of control of extraneous variables (e.g., time of day, noise, sitting distance, etc.). Thus, it will be important to replicate the present findings with a specified control group both in terms of diagnostic features as well as the sampling approach.

Finally, there are limitations due to characteristics of the procedure. Our materials depicted men exclusively and were pretested with individuals with unknown BPD status, and we used colored backgrounds as contextual cues; it is therefore, at this point, not yet possible to generalize our results to the presentation of women´s faces, or to more natural, ecologically valid (and potentially more salient) contexts. We also only used the (neutral) faces of others rather than voices or other modalities to update impressions, which has been shown to be more effective [66]. Future studies might also consider using interim evaluations between exposures to different contexts. The adapted impression formation task has previously been used without interim evaluations [29]. Although one could argue that interim evaluations could demarcate a context change or change evaluative strategies [50, 51], this assumption has not been tested directly for the specific impression formation task, and interim evaluations could allow to determine if there are overall differences in the initial learning processes for individuals with BPD, or whether individuals with BPD could be more prone to base evaluations on more recently presented information. This would also help to determine if individuals with BPD reacted more or less strongly to different kinds of evaluative statements (i.e., self- vs. other-relevant), which could partly explain the more pronounced updating in the self-relevant condition. In a similar vein, future studies might include a control condition that presents consistent information across contexts to estimate overall differences in impression formation. Therefore, replicating the present findings in more diverse samples and using more ample and inclusive materials is essential.

## Conclusion

The present study suggests novel insights into the cognitive processes underlying interpersonal difficulties in BPD, highlighting alterations in impression formation and updating as potential mechanisms contributing to the phenomenology of the disorder. By elucidating these cognitive mechanisms, this research could advance our understanding of BPD and inform the development of targeted interventions to improve interpersonal relationships and social functioning in individuals with BPD.

### Electronic supplementary material

Below is the link to the electronic supplementary material.

Supplementary Material 1

Supplementary Material 2

## Data Availability

The datasets and materials of the current study are available from the corresponding author on reasonable request.

## Footnotes

1 We originally registered a repeated-measures ANOVA with context as an additional repeated-measures factor, but switched to a MANOVA-based analysis out of concern for unanticipated sphericity violations, Mauchly’s *W* = 0.9, *p* = .01, and the MANOVAs increased power for detecting small reliable changes between highly correlated conditions [67]. However, the planned analysis’ pairwise comparisons produced inferentially identical results, see Additional file 2.

## Declarations

### Author information

KK performed this work in fulfillment of the requirements for obtaining the degree “Dr. med.”.

## Abbreviations

BPD: Borderline Personality Disorder
BSL-23: Borderline Symptom List
RSQ-9: Rejection Sensitivity Questionnaire
