# Supplementary material for: A second chance for first impressions: evidence for altered impression updating in borderline personality disorder
Source: Borderline Personal Disord Emot Dysregul. 2024 Jul 18;11:15. doi: 10.1186/s40479-024-00259-y (PMC11256375; doi:10.1186/s40479-024-00259-y)
Supplement: Supplementary file 1 — Supplementary Material 1 [file 40479_2024_259_MOESM1_ESM.docx]

**Statement Pre-Testing**

We conducted a pre-test to select positive and negative statements with similar evaluations across self-relevant and other-relevant versions, respectively. N = 50 individuals (42 women, 8 men, *M*_age_ = 27.5, age range: 18 – 58 years) participated in an online survey. After providing sociodemographic information and completing the BSL-23 (1) and RSQ-9 questionnaires (2,3), each participant rated the valence of different behavioral statements on a visual analogue scale (VAS) with a range of -100 (negative) to 100 (positive), with the neutral midpoint (0) serving as a starting point for each rating. The statements were selected from a pool of 40 positive and 40 negative statements, adapted from Rydell and Gawronski (4), for which we designed parallel self-relevant and other-relevant versions. For example, one positive item either stated “a person offers to help you without being asked” in the self-relevant version, or “a person offers to help a friend without being asked” in the other-relevant version. We randomly selected ten statements per each 2 (valence: positive vs. negative) × 2 (relevance: self vs. other) categories such that a participant would only see one of the parallel versions (i.e., behaviors were not repeated within participants). We additionally included ten positive and ten negative filler statements without a social referent as control stimuli (e.g., “a person is committed to animal welfare”), such that there were 60 statements in total. The procedure took about 15 min to be complete.

We based the item selection on the results of a linear-mixed model of ratings with relevance, behavior, and their interaction as fixed effects, and a by-participant random intercept. The model was computed with *R* version 4.3.2 (5) package *lme4* version 1.1.35.1 (6), and simple contrasts via *emmeans* version 1.10.0 (7) were used to evaluate statistical difference between self- and other-relevant versions of behaviors. We selected the 16 positive and 16 negative behaviors among the non-significant comparisons with the lowest standardized mean difference between self- and other-relevant versions (see **Tables S1 and S2**; for original-language German versions, see **Table S3**).

To confirm the adequacy of the item selection, we averaged the ratings on a per-participant basis for the 2 (valence: positive vs. negative) × 2 (relevance: self vs. other) behavior categories, and submitted these means to an equivalent 2 × 2 repeated-measures ANOVA. Consistent with the intended selection, statement ratings varied by valence, *F*(1, 48) = 567.16, *p* < .001, *η²* = .92, with positive behaviors receiving more favorable evaluations (*M_positive_* = 69.48, *SD* = 22.58 vs. *M_negative_* = -50.66, *SD* = 17.90). Neither the relevance main effect nor the valence × relevance interaction were significant, *F*s < 0.1, *p*s > .81, suggesting comparability of the parallel versions. In addition, we computed bi-variate correlations of the rating means within each category with the BSL-23 and RSQ-9 scores, which provided no indication that behavior ratings varied based on BPD psychopathology or rejection sensitivity scores, all *p*s > .13.

**References**

1. Bohus M, Kleindienst N, Limberger MF, Stieglitz RD, Domsalla M, Chapman AL, et al. The short version of the Borderline Symptom List (BSL-23): Development and initial data on psychometric properties. Psychopathology [Internet]. 2009 Jan [cited 2021 Mar 22];42(1):32–9. Available from: https://pubmed.ncbi.nlm.nih.gov/19023232/

2. Staebler K, Helbing E, Rosenbach C, Renneberg B. Rejection sensitivity and borderline personality disorder. Clin Psychol Psychother [Internet]. 2011 [cited 2021 Mar 22];18(4):275–83. Available from: http://socialrelations.psych.

3. Berenson KR, Gyurak A, Ayduk Ö, Downey G, Garner MJ, Mogg K, et al. Rejection sensitivity and disruption of attention by social threat cues. J Res Pers [Internet]. 2009 Dec 12 [cited 2024 Jan 25];43(6):1064–72. Available from: /pmc/articles/PMC2771869/

4. Rydell RJ, Gawronski B. I like you, I like you not: Understanding the formation of context-dependent automatic attitudes. Cogn Emot [Internet]. 2009 [cited 2021 Mar 22];23(6):1118–52. Available from: www.psypress.com/cogemotion

5. R Core Team. R: A Language and Environment for Statistical Computing [Internet]. Vienna, Austria; 2022. Available from: https://www.r-project.org/

6. Bates D, Mächler M, Bolker BM, Walker SC. Fitting Linear Mixed-Effects Models Using lme4. J Stat Softw [Internet]. 2015 Oct 7 [cited 2022 Apr 6];67(1):1–48. Available from: https://www.jstatsoft.org/index.php/jss/article/view/v067i01

7. Lenth R V. emmeans: Estimated Marginal Means, aka Least-Squares Means [Internet]. 2022. Available from: https://cran.r-project.org/package=emmeans

**Table S1**

*Positive item valence ratings means (M), standard deviations (SD), and number of ratings (n).*

| **self-relevant** | **n** | **M** | **SD** | **other-relevant** | **n** | **M** | **SD** |
| --- | --- | --- | --- | --- | --- | --- | --- |
| reminds you of an important appointment. | 18 | 72.67 | 37.46 | reminds a colleague of an important appointment. | 12 | 65.83 | 31.96 |
| gives you a lift when you have to go to an important appointment. | 13 | 74.38 | 26.70 | takes someone who has to go to an important appointment. | 12 | 80.00 | 26.60 |
| likes to ask you for advice. | 15 | 53.07 | 42.57 | likes to ask others for advice. | 11 | 50.45 | 43.44 |
| buys you a drink in the pub. | 10 | 51.60 | 37.34 | buys everyone a round in the pub. | 12 | 54.67 | 31.83 |
| waters the plants for you during your vacation. | 11 | 75.64 | 29.96 | waters the plants for others while on vacation. | 10 | 83.90 | 21.89 |
| helps you with an important question. | 13 | 80.38 | 21.48 | helps a colleague with an important question. | 13 | 82.85 | 26.35 |
| helps you fix the tire on your bike. | 11 | 80.00 | 30.38 | helps a friend fix the tire on her bike. | 14 | 71.79 | 33.68 |
| helps you renovate. | 17 | 81.65 | 27.09 | helps a friend with renovations. | 15 | 81.13 | 20.02 |
| lends you money when you are short of it. | 12 | 62.00 | 35.15 | lends money to friends when money is tight. | 8 | 58.88 | 43.17 |
| provides first aid when you have an accident. | 10 | 85.40 | 31.52 | gives first aid when someone else has an accident. | 10 | 81.70 | 29.80 |
| pays you a compliment. | 10 | 57.10 | 33.71 | compliments a friend. | 14 | 55.07 | 36.24 |
| always makes time for you. | 16 | 73.00 | 42.06 | always makes time for others. | 8 | 80.38 | 23.57 |
| travels across the country to support you in a difficult time. | 12 | 83.00 | 33.06 | travels across the country to support a friend in a difficult time. | 14 | 74.07 | 32.42 |
| repairs an old guitar for you. | 13 | 68.62 | 27.30 | repairs an old guitar for a neighbor. | 11 | 74.91 | 28.20 |
| gives you something nice for Valentine's Day. | 9 | 65.33 | 34.11 | gives his partner something nice for Valentine's Day. | 12 | 68.08 | 34.01 |
| always says 'Good morning' to you and smiles. | 7 | 53.00 | 46.69 | always says 'Good morning' to others and smiles. | 19 | 54.42 | 35.68 |

*Note*. All statements were preceded with “A person…”. Ratings provided on a visual analogue scale (VAS) ranged from -100 (negative) to 100 (positive).

**Table S2**

*Negative item valence ratings means (M), standard deviations (SD), and number of ratings (n).*

| **self-relevant** | **n** | **M** | **SD** | **other-relevant** | **n** | **M** | **SD** |
| --- | --- | --- | --- | --- | --- | --- | --- |
| treats your things badly. | 11 | -80.91 | 29.63 | treats other people's things badly. | 11 | -94.55 | 6.65 |
| ignores you even though they know each other. | 14 | -47.21 | 39.06 | ignores a colleague even though they know each other. | 15 | -59.40 | 36.81 |
| laughs when you tell a sad story. | 16 | -59.56 | 35.44 | laughs when someone else tells a sad story. | 11 | -68.73 | 34.11 |
| walks past you and does not say hello. | 11 | -34.73 | 33.43 | walks past others and does not greet them. | 14 | -21.00 | 23.21 |
| borrows things from you and doesn't give them back. | 12 | -74.00 | 30.12 | borrows things from others and does not give them back. | 11 | -87.36 | 24.48 |
| wants you to be fired if you are late. | 15 | -82.80 | 32.08 | wants colleagues to be fired if they are late. | 10 | -68.90 | 30.31 |
| gets upset if you don't agree with them. | 10 | -44.10 | 29.18 | gets upset when others disagree with them. | 16 | -46.81 | 39.28 |
| tells you that she has no time to help. | 11 | -19.27 | 26.31 | tells others that she has no time to help. | 11 | -26.64 | 33.76 |
| declines your invitation because she has other plans. | 10 | -28.00 | 20.92 | cancels someone's invitation because she has other plans. | 10 | -19.70 | 38.10 |
| cancels a meeting with you at short notice. | 11 | -47.18 | 36.61 | cancels a meeting with someone else at short notice. | 13 | -39.62 | 28.94 |
| yells at you in public. | 7 | -73.86 | 38.29 | yells at her partner in public. | 13 | -78.46 | 33.78 |
| doesn't understand the joke you made. | 17 | -14.82 | 33.08 | doesn't understand someone else's joke. | 18 | -11.50 | 23.41 |
| plans a vacation without you. | 9 | -11.00 | 22.12 | plans a vacation without her partner. | 17 | -8.65 | 27.62 |
| insists on being addressed by you. | 12 | -35.17 | 32.62 | insists on being addressed by younger people. | 11 | -46.91 | 38.50 |
| throws your vase on the floor in anger. | 15 | -94.27 | 8.32 | throws a friend's vase on the floor in anger. | 10 | -82.00 | 24.93 |
| calls in sick at your work to take time off. | 13 | -62.92 | 41.14 | calls in sick at work to take time off. | 14 | -61.21 | 36.59 |

*Note*. All statements were preceded with “A person…”. Ratings provided on a visual analogue scale (VAS) ranged from -100 (negative) to 100 (positive).

**Table S3**

*Original-language German item versions.*

| **Self-relevant** | **Other-relevant** |
| --- | --- |
|  |  |
| *Positive Items* | |
|  | |
| erinnert Sie an einen wichtigen Termin. | erinnert einen Kollegen an einen wichtigen Termin. |
| nimmt Sie mit, als Sie zu einem wichtigen Termin müssen. | nimmt jemanden mit, der zu einem wichtigen Termin muss. |
| fragt Sie gerne um Rat. | fragt andere gerne um Rat. |
| gibt Ihnen in der Kneipe eine Runde aus. | gibt in der Kneipe allen eine Runde aus. |
| gießt für Sie die Pflanzen während des Urlaubs. | gießt für andere die Pflanzen während des Urlaubs. |
| hilft Ihnen bei einer wichtigen Frage weiter. | hilft einer Kollegin bei einer wichtigen Frage weiter. |
| hilft Ihnen, den Reifen am Fahrrad zu reparieren. | hilft einer Bekannten, den Reifen am Fahrrad zu reparieren. |
| hilft Ihnen beim Renovieren. | hilft einem Bekannten beim Renovieren. |
| leiht Ihnen Geld, wenn es gerade knapp ist. | leiht Freunden Geld, wenn es gerade knapp ist. |
| leistet erste Hilfe, als Sie einen Unfall haben. | leistet erste Hilfe, als jemand anderes einen Unfall hat. |
| macht Ihnen ein Kompliment. | macht einer Bekannten ein Kompliment. |
| nimmt sich immer Zeit für Sie. | nimmt sich immer Zeit für andere. |
| reist einmal quer durchs Land, um Sie in einer schweren Zeit zu unterstützen. | reist einmal quer durchs Land, um einen Freund in einer schweren Zeit zu unterstützen. |
| repariert eine alte Gitarre für Sie. | repariert eine alte Gitarre für einen Nachbarn. |
| schenkt Ihnen etwas Schönes zum Valentinstag. | schenkt der Partnerin etwas Schönes zum Valentinstag. |
| sagt Ihnen immer 'Guten Morgen' und lächtelt dabei. | sagt anderen immer 'Guten Morgen' und lächtelt dabei. |
|  |  |
| *Negative Items* | |
|  | |
| behandelt die Sachen von Ihnen schlecht. | behandelt die Sachen von anderen schlecht. |
| ignoriert Sie, obwohl sie sich kennen. | ignoriert eine Kollegin, obwohl sie sich kennen. |
| lacht, als Sie eine traurige Geschichte erzählen. | lacht, als jemand anderes eine traurige Geschichte erzählt. |
| läuft an Ihnen vorbei und grüßt nicht. | läuft an anderen vorbei und grüßt nicht. |
| leiht sich Sachen von Ihnen und gibt sie nicht zurück. | leiht sich Sachen von anderen und gibt sie nicht zurück. |
| möchte das Sie, wenn sie zu spät kommen, gefeuert werden. | möchte das Kollegen, wenn sie zu spät kommen, gefeuert werden. |
| regt sich auf, wenn Sie nicht der gleichen Meinung sind. | regt sich auf, wenn andere nicht der gleichen Meinung sind. |
| sagt Ihnen, dass Sie keine Zeit zum helfen hat. | sagt anderen, dass sie keine Zeit zum helfen hat. |
| sagt die Einladung von Ihnen ab, weil sie andere Pläne hat. | sagt die Einladung von jemanden ab, weil sie andere Pläne hat. |
| sagt ein Treffen mit Ihnen kurzfristig ab. | sagt ein Treffen mit jemand anderem kurzfristig ab. |
| schreit Sie in der Öffentlichkeit an. | schreit den Partner in der Öffentlichkeit an. |
| versteht den Witz von Ihnen nicht. | versteht den Witz von jemand anderem nicht. |
| plant einen Urlaub ohne Sie. | plant einen Urlaub ohne die eigene Partnerin. |
| besteht darauf, von Ihnen gesiezt zu werden. | besteht darauf, von jüngeren gesiezt zu werden. |
| wirft vor Wut die Vase von Ihnen auf den Boden. | wirft vor Wut die Vase einer Freundin auf den Boden. |
| meldet sich auf Ihrer Arbeit krank, um frei zu machen. | meldet sich auf der Arbeit krank, um frei zu machen. |

*Note*. All statements were preceded with “Eine Person…”.
