# Supplementary material for: A second chance for first impressions: evidence for altered impression updating in borderline personality disorder
Source: Borderline Personal Disord Emot Dysregul. 2024 Jul 18;11:15. doi: 10.1186/s40479-024-00259-y (PMC11256375; doi:10.1186/s40479-024-00259-y)
Supplement: Supplementary file 2 — Supplementary Material 2 [file 40479_2024_259_MOESM2_ESM.docx]

**Additional Analyses**

**Repeated-measures ANOVA**

We originally registered a repeated-measures ANOVA with context as an additional repeated-measures factor as our primary analysis, but switched to a MANOVA-based analysis out of concern for unanticipated sphericity violations when including context as a three-leveled factor, Mauchly's *W* = 0.9, *p* = .01, and because the MANOVAs has increased power for detecting small reliable changes between highly correlated conditions (1). Here, were report the result of the 2 (group: BPD vs. controls) × 2 (relevance: self vs. other) × 2 (initial valence: positive vs. negative) x 3 (Context: A vs. B. vs. C) ANOVA, with valence and context as repeated-measures factors, and the Bonferroni-corrected planned comparisons between valence conditions in the four-way interaction.

The overall ANOVA found a significant main effect of valence, *F*(1, 88) = 13.60, *p* < .001, *η²* = .13, group, *F*(1, 88) = 13.81, *p* < .001, *η²* = .14, a valence × group interaction, *F*(1, 88) = 18.50, *p* < .001, *η²* = .17, and a valence × group × relevance interaction, *F*(1, 88) = 4.84, *p* = .03, *η²* = .05. The valence × group × relevance × context interaction was not significant, *F*(1, 88) = 1.51, *p* = .22, *η²* = .02. Planned comparisons between valence conditions within the other factor combinations showed that controls evaluated faces in contexts A, B, and C consistent with the valence of the initial statements in the self-relevant condition, *p*s < .002, and in contexts A and C in the other-relevant condition, *p*s < .05. The preference on Context B was not significant, *p* = .36. Individuals with BPD preferred the faces initially paired with negative statements over initially positively paired faces in the self-relevant condition in Context C, *p =* .04, and there were no significant differences in the other conditions, all *p*s > .39.

**Linear Mixed Model**

Because we assessed BPD psychopathology dimensionally using the BSL-23, we also re-analyzed our data with BPD as a continuous rather than categorical predictor. Specifically, we fitted a linear mixed model to the data with BSL scores (mean centered), Valence, Relevance, Context, and all interactions as fixed effects, and by-participant random intercepts (and random slopes, if justified by the data). Mixed models were fitted using restricted maximum likelihood (REML) estimation in R package lme4 Version 1.1.35.1 (2). The R version used was 4.3.3 (3).

A model with all fixed effects fit the data better compared to a baseline model with only a by-participant random intercept, χ² (df = 23) = 79.12, *p* < .001. The model fit improved further by including a by-participant random slope for Valence, χ² (df = 2) = 40.31, *p* < .001, but not when including a random slope for the effect of context, *p* = .98. The models did not converge when including a random slope for the effect of Relevance.

The final model’s results are summarized in **Table S4**. The analysis revealed a significant effect for Valence, a BSL × Valence interaction, and the predicted BSL × Valence × Relevance × Context interaction. Similar to the primary analysis, the individual evaluation of the model estimates (**Table S5**), using 95% confidence intervals not including 0, showed that more pronounced BSL scores were associated with reduced effects of valence in contexts C and B in the self-relevant conditions (see the last two rows). The analysis code is shown in **Table S6**.

**Table S4**

*Type-III analysis-of-variance table for the mixed model.*

| **Predictor** | **χ²** | **df** | **p-value** |
| --- | --- | --- | --- |
| BSL | 0.05 | 1 | .830 |
| Valence | 394.13 | 2 | <.001 |
| Relevance | 0.51 | 1 | .477 |
| Context | 0.88 | 2 | .644 |
| BSL × Valence | 5.04 | 1 | .025 |
| BSL × Relevance | 0.20 | 1 | .659 |
| Valence × Relevance | 0.01 | 1 | .940 |
| BSL × Context | 1.23 | 2 | .539 |
| Valence × Context | 2.61 | 2 | .271 |
| Relevance × Context | 0.16 | 2 | .923 |
| BSl × Valence × Relevance | 0.22 | 1 | .636 |
| BSL × Valence × Context | 5.53 | 2 | .063 |
| BSL × Relevance × Context | 1.01 | 2 | .605 |
| Valence × Relevance × Context | 0.34 | 2 | .843 |
| BSL × Valence × Relevance × Context | 6.43 | 2 | .040 |

**Table S5**

*Mixed model fixed effect estimates with 95% confidence intervals (CI).*

| **Predictor** | **B** | **SE** | **t** | **LL** | **UL** |
| --- | --- | --- | --- | --- | --- |
| BSL | -1.15 | 5.37 | -0.22 | -11.49 | 9.18 |
| Valence(neg) | 68.08 | 5.34 | 12.74 | 57.80 | 78.37 |
| Valence(pos) | 88.95 | 5.44 | 16.34 | 78.47 | 99.43 |
| Relevance(self) | -5.62 | 7.91 | -0.71 | -20.84 | 9.60 |
| Context(B) | 3.27 | 5.82 | 0.56 | -7.93 | 14.46 |
| Context(C) | 5.43 | 5.82 | 0.93 | -5.76 | 16.62 |
| BSL × Valence(pos) | -16.39 | 7.30 | -2.25 | -30.43 | -2.34 |
| BSL × Relevance(self) | 3.17 | 7.17 | 0.44 | -10.64 | 16.97 |
| Valence(pos) × Relevance(self) | 0.81 | 10.75 | 0.08 | -19.87 | 21.50 |
| BSL × Context(B) | -5.81 | 5.85 | -0.99 | -17.05 | 5.44 |
| BSL × Context(C) | -0.37 | 5.85 | -0.06 | -11.61 | 10.88 |
| Valence(pos) × Context(B) | -13.15 | 8.24 | -1.60 | -28.98 | 2.68 |
| Valence(pos) × Context(C) | -8.39 | 8.24 | -1.02 | -24.22 | 7.44 |
| Relevance(self) × Context(B) | 3.45 | 8.62 | 0.40 | -13.11 | 20.02 |
| Relevance(self) × Context(C) | 1.64 | 8.62 | 0.19 | -14.93 | 18.20 |
| BSL × Valence(pos) × Relevance(self) | 4.61 | 9.75 | 0.47 | -14.15 | 23.37 |
| BSL × Valence(pos) × Context(B) | 19.43 | 8.28 | 2.35 | 3.53 | 35.34 |
| BSL × Valence(pos) × Context(C) | 10.66 | 8.28 | 1.29 | -5.25 | 26.56 |
| BSL × Relevance(self) × Context(B) | 7.07 | 7.82 | 0.91 | -7.95 | 22.09 |
| BSL × Relevance(self) × Context(C) | 0.61 | 7.82 | 0.08 | -14.41 | 15.63 |
| Valence(pos) × Relevance(self) × Context(B) | 5.29 | 12.19 | 0.43 | -18.14 | 28.72 |
| Valence(pos) × Relevance(self) × Context(C) | -1.47 | 12.19 | -0.12 | -24.90 | 21.96 |
| BSL × Valence(pos) × Relevance(self) × Context(B) | -23.61 | 11.05 | -2.14 | -44.86 | -2.37 |
| BSL × Valence(pos) × Relevance(self) × Context(C) | -24.87 | 11.05 | -2.25 | -46.12 | -3.63 |

*Note*. B = regression weight, LL = lower level CI, UL = upper level CI.

**Table S6**

*Mixed model R Code*

| #loading packages  library(lme4)  library(car)  #loading data  dat=read.table("aggr_lf.csv",sep=";",header=TRUE)  head(dat)  #estimate mixed models  mod1=lmer(eval2~1+(1\|id),data=dat)  mod2=lmer(eval2~bsl_cen*factor(valence)*factor(condition_social)*factor(context)-1+(1\|id),data=dat)  mod3=lmer(eval2~bsl_cen*factor(valence)*factor(condition_social)*factor(context)-1+(val\|id),data=dat)  mod4=lmer(eval2~bsl_cen*factor(valence)*factor(condition_social)*factor(context)-1+(val+context\|id),data=dat)  mod5=lmer(eval2~bsl_cen*factor(valence)*factor(condition_social)*factor(context)-1+(val+condition_social\|id),data=dat)  #model selection  anova(mod1,mod2,refit=TRUE)  anova(mod2,mod3,refit=FALSE)  anova(mod3,mod4,refit=FALSE)  #model evaluation  summary(mod3)  Anova(mod3,type="III")  #compute CIs with lmerTest  library(lmerTest)  mod3=lmer(eval2~bsl_cen*factor(valence)*factor(condition_social)*factor(context)-1+(val\|id),data=dat)  confint(mod3, parm="beta_", level = 0.95) |
| --- |

**References**

1. Field A. A bluffer’s guide to sphericity. Br Psychol Soc Math Stat \& Comput Sect Newsl. 1998;6(1):13–22.

2. Bates D, Mächler M, Bolker BM, Walker SC. Fitting Linear Mixed-Effects Models Using lme4. J Stat Softw [Internet]. 2015 Oct 7 [cited 2022 Apr 6];67(1):1–48. Available from: https://www.jstatsoft.org/index.php/jss/article/view/v067i01

3. R Core Team. R: A Language and Environment for Statistical Computing [Internet]. Vienna, Austria; 2022. Available from: https://www.r-project.org/
